# Possible role of Krüppel-like factor 5 in the remodeling of small airways and pulmonary vessels in chronic obstructive pulmonary disease

**DOI:** 10.1186/s12931-016-0322-y

**Published:** 2016-01-20

**Authors:** Kyoko Abe, Hisatoshi Sugiura, Yuichiro Hashimoto, Tomohiro Ichikawa, Akira Koarai, Mitsuhiro Yamada, Tadahisa Numakura, Katsuhiro Onodera, Rie Tanaka, Kei Sato, Satoru Yanagisawa, Tatsuma Okazaki, Tsutomu Tamada, Toshiaki Kikuchi, Masakazu Ichinose

**Affiliations:** Department of Respiratory Medicine, Tohoku University Graduate School of Medicine, 1-1 Seiryo-machi, Aoba-ku, Sendai, 980-8574 Japan; Third Department of Internal Medicine, Wakayama Medical University, School of Medicine, 811-1 Kimiidera, Wakayama, 641-8509 Japan; Department of Respiratory Medicine and Infectious Diseases, Niigata University Graduate School of Medical and Dental Sciences, 1-757 Asahimachidori, Chuo-ku, Niigata 951-8510 Japan

**Keywords:** Fibroblasts, Oxidative stress, Nitrosative stress, Metalloproteases, Disease susceptibility

## Abstract

**Background:**

Small airway remodeling is an important cause of the airflow limitation in chronic obstructive pulmonary disease (COPD). A large population of patients with COPD also have pulmonary hypertension. Krüppel-like factor 5 (KLF5) is a zinc-finger transcription factor that contributes to tissue remodeling in cardiovascular diseases. Here, we evaluate the possible involvement of KLF5 in the remodeling of small airways and pulmonary vessels in COPD.

**Methods:**

Lung tissues were obtained from 23 control never-smokers, 17 control ex-smokers and 24 ex-smokers with COPD. The expression of KLF5 in the lung tissues was investigated by immunohistochemistry. We investigated whether oxidative/nitrosative stress, which is a major cause of the pathogenesis in COPD, could augment the production of KLF5. We examined the role of KLF5 in the stress-mediated tissue remodeling responses. We also investigated the susceptibility of KLF5 expression to nitrosative stress using bronchial fibroblasts isolated from the lung tissues.

**Results:**

The expression of KLF5 was up-regulated in the small airways and pulmonary vessels of the COPD patients and it was mainly expressed in bronchial fibroblasts and cells of the pulmonary vessels. The extent of the KLF5 expression in the small airway of the COPD group had a significant correlation with the severity of the airflow limitation. Oxidative/nitrosative stress augmented the production of KLF5 in lung fibroblasts as well as the translocation of KLF5 into the nuclei. Silencing of KLF5 suppressed the stress-augmented differentiation into myofibroblasts, the release of collagens and metalloproteinases. Bronchial fibroblasts from the patients with COPD highly expressed KLF5 compared to those from the control subjects under basal condition and were more susceptible to the induction of KLF5 expression by nitrosative stress compared to those from the control subjects.

**Conclusion:**

We provide the first evidence that the expression of KLF5 is up-regulated in small airways and pulmonary vessels of patients with COPD and may be involved in the tissue remodeling of COPD.

## Background

The airflow limitation of chronic obstructive pulmonary disease (COPD) patients is thought to be result from two main pathological changes [1]. One is obstruction of the small conducting airways by inflammatory mucous exudates and airway wall remodeling, and the other is the loss of elastic recoil because of emphysematous destruction of the pulmonary parenchyma [1–3]. Persistent inflammation and oxidative/nitrosative stress cause remodeling of the small airways. Some previous reports showed that the hyperproduction of growth factors such as transforming growth factor-β_1_ (TGF-β_1_) and matrix metalloproteinases (MMPs) could be involved in the remodeling of the small airways [4–8]. Other reports showed that MMPs degrade extracellular matrix (ECM) proteins, participate in ECM turnover, and promote subsequent structural changes of the airways [9–11]. Although the remodeling of small airways and pulmonary vessels is apparent in COPD lungs and molecules such as TGF-β_1_ and MMPs could be involved in the tissue remodeling, the precise mechanisms responsible for the tissue remodeling remain poorly understood.

Oxidative stress has an important role in accelerating the deterioration of lung function in COPD (1). Indeed, the production of reactive oxygen species (ROS) such as hydrogen peroxide (H_2_O_2_) is enhanced in the airways of COPD patients [12–14]. Oxidative stress activates inflammatory genes, inactivates antiproteases and stimulates mucus secretion [15, 16]. According to the Global Initiative for Chronic Obstructive Lung Disease (GOLD) guidelines, many of these adverse effects are mediated by reactive nitrogen species (RNS) such as peroxynitrite (ONOO^−^). We previously demonstrated that the production of RNS was augmented in the airways of COPD patients compared to healthy subjects or asthmatic patients [17]. Both ROS and RNS are reported to stimulate tissue remodeling through nuclear factor-κB (NF-κB) or activator protein-1 (AP-1) *in vitro* [18–20]. However, another transcription factor responsible for remodeling of the small airways and pulmonary vessels in COPD has not been elucidated yet.

KLF5 is a zing finger transcription factor that belongs to the family of Krüppel-like factors and is implicated in important biological functions, including embryonic development, cellular differentiation and proliferation [21–23]. Although a member of the KLF family such as KLF2 is reported to be involved in the development of various organs, especially lung development, KLF5 is thought to be a key trnacription factor in tissue remodeling among the KLF family [21–26]. The expression of KLF5 is activated in cardiac fibroblasts and contributes to tissue remodeling in cardiovascular diseases [24, 25]. Although KLF5 is a key player in the tissue remodeling of various organs [24–26], its role in the remodeling of small airways and pulmonary vessels in COPD remains unclear.

The present study was designed first to determine whether the expression of KLF5 is up-regulated in the lungs of COPD patients. Second, we investigated whether ROS or RNS augment the expression and activation of KLF5 in fibroblasts and whether KLF5 regulates the tissue remodeling responses. Third, we examined the susceptibility of KLF5 overexpression induced by nitrosative stress in bronchial fibroblasts from COPD patients.

## Methods

### Preparation of human lung tissues

Twenty-three control never-smokers, 17 control ex-smokers and 24 ex-smokers with COPD took part in the study after giving written informed consent. COPD was diagnosed according to the GOLD guidelines [1]. Because lung cancer is known to stimulate the expression of KLF5, all the study subjects had had lung cancer and peripheral lung tissues were obtained from the subpleural parenchyma of the lobe resected at surgery, avoiding areas involved by tumors. All subjects had undergone surgical operation for lung cancer after receiving pulmonary function tests. The tissues were used for immunohistostaining and the culture of bronchial fibroblasts. All experiments in the current study were approved by the ethics committee of Tohoku University Graduate School of Medicine.

### Immunochemical detection of KLF5 and prolyl 4-hydroxylase β (P4HB)

The lung tissues from the control never-smokers, control ex-smokers and ex-smokers with COPD were used in the immunohistological examination of KLF5 and P4HB. Briefly, after fixation with 4 % paraformaldehyde for 30 min, the tissues were blocked with 1 % skim milk for 30 min and rinsed. The samples were incubated with rabbit polyclonal anti-KLF5 antibody (1:1000 dilution, Abcam, Cambridge, UK), plus mouse monoclonal anti-P4HB antibody (1:400 dilution, Novus, Littleton, CO), or non-specific polyclonal rabbit IgG as a negative control at 4 °C overnight. After washing for immunohistochemistry, the samples were treated with the goat anti-rabbit IgG horseradish peroxidase labeled polymer-conjugated secondary antibody (Santa Cruz Biotechnology, Dallas, TX) for 60 min and 3,3′-diaminobenzidine (Nichirei Biosciences Inc, Tokyo, Japan) was used to visualize labeling. The samples were viewed by microscopy (BX53-33-SDO, Olympus, Tokyo, Japan) and photographed with a digital camera (DP71-SET, Olympus, Tokyo, Japan). For immunofluorescent staining, the samples were incubated with goat anti-rabbit IgG conjugated with fluorescein isothiocyanate (FITC, 1:1000 dilution, Abcam) for 60 min at room temperature. Then, after washing again, they were incubated with goat anti-mouse IgG conjugated with Dylight 650 (1:50 dilution, Abcam) for 60 min. After washing, they were stained with Fluoromount-G containing DAPI (Southern Biotech, Birmingham, AL) and then viewed and photographed using a multiphoton confocal LSM 780 NLO microscope system (Carl Zeiss, Jena, Germany).

### Measurement of KLF5 positive cells

The KLF5-positive area was quantified as follows. Three areas per each lung specimen were randomly chosen. One area usually contained a couple of peripheral airways. Among these areas, five random airways were chosen by two investigators without any prior knowledge of the background of the subjects. The area of KLF5 in small airways was defined as the submucosal area of KLF5-positive cells standardized by the length of the airway basement membrane. The area of KLF5 in pulmonary small vessels was also defined as the vascular wall area of KLF5-positive cells standardized by the lumen diameter of the pulmonary vessels. The area and the length were measured using Image J (National Institutes of Health, Frederick, MD).

### Cell culture

Human fetal lung fibroblasts (HFL-1) were obtained from the American Type Culture Collection (Rockville, MD). Four different strains of adult fibroblasts were obtained from lung tissues resected by surgical operation from COPD or non-COPD patients with lung cancer in our institution. The HFL-1 and adult cells were cultured on tissue culture dishes with Dulbecco’s Modified Eagle’s Medium (DMEM, Invitrogen Life Technologies, Grand Island, NY) supplemented with 10 % fetal calf serum (FCS, Invitrogen Life Technologies), 100 unit/ml penicillin, and 100 μg/ml streptomycin. Cells were cultured at 37 °C in a humidified atmosphere of 5 % CO_2_ and passaged. HFL-1 cells were used between the 16th and 18th passages. The adult bronchial fibroblasts from the study subjects were used between the 4th-5th passages.

### Western blotting

Cells were seeded in 60 mm dishes at a density of 1 × 10^5^ /ml. At 90 % confluence, the cells were treated with various concentrations of H_2_O_2_ (Wako, Osaka, Japan) or ONOO^−^ (Calbiochem, La Jolla, CA). To obtain the nuclear fractions, a Nuclear Extraction Kit (Active Motif, Carlsbad, CA) was used according to the manufacturer’s instructions. The cells were washed with ice-cold PBS and homogenized in cell lysis buffer. Equal amounts of protein were loaded and separated by electrophoresis on 12 % SDS polyacrylamide gels. The separated proteins were transferred to a polyvinylidene difluoride membrane (Bio-Rad Laboratories, Hercules, CA). The following antibodies were used for detection of the target proteins: rabbit polyclonal anti-KLF5 antibody (1:1000 dilution, Abcam), mouse monoclonal anti-β-actin antibody (1:4000 dilution, Sigma-Aldrich, St. Louis, MO), mouse monoclonal anti-lamin A/C antibody (1:400 dilution, Santa Cruz Biotechnology, Dallas, TX), mouse monoclonal anti-α-SMA (1:5000 dilution, Sigma-Aldrich). Bound antibodies were visualized using the appropriate peroxidase-conjugated secondary antibodies and enhanced chemiluminescence (GE Healthcare Life Sciences, Buckinghamshire, UK) with a chemiluminescene imaging system (LAS-4000 mini, Fujifilm, Tokyo, Japan). Band intensity was quantified by densitometry (Quantity One, Bio-Rad Laboratories).

### Silencing of KLF5

Small interfering RNA (siRNA) for *KLF5* (ON-TARGETplus SMART pool siRNA) and control siRNA (ON-TARGET plus Non-targeting Pool) were purchased from Darmacon (Lafayette, CO). Using Lipofectamine RNAiMAX (Invitrogen Life Technologies), the siRNA was transfected into HFL-1 cells plated in 60 mm dishes at a final concentration of 5 nM in 5 ml of 10 % FCS DMEM without antibiotics. The infected cells were used for the experiments of α-smooth muscle actin production and release of collagen and MMPs.

### Quantitative reverse transcription-polymerase chain reaction (qRT-PCR)

Total RNA was isolated from homogenized HFL-1 cells using RNeasy kit (Qiagen, Valencia, CA). RNA samples were reverse-transcribed with a High Capacity RNA-to-cDNA Kit (Applied Biosystems, Forster City, CA). qRT-PCR was performed using Power SYBR Green PCR Master Mix (Applied Biosystems Life Technologies, Warrington, UK), and was carried out on Step One Plus (Applied Biosystems). The data were standardized by GAPDH. The primer sequences were as follows: *KLF5*, 5′-ACGACGCATCCACTACTGCG-3′ and 5′-GTGAGTCCTCAGGTGAGCTT-3′; *GAPDH*, 5′-GCACCGTCAAGGCTGAGAAC-3′ and 5′-TGGTGAAGACGCCAGTGGA-3′.

### Collagen assay

The amounts of collagen in the supernatants were measured using the Sircol Collagen Assay kit (Biocolor Ltd., Belfast, Northern Ireland) according to the manufacturer’s protocol.

### Zymogram

Supernatants from the cell cultures (500 μl per culture condition) were concentrated 10-fold by precipitation with cold ethanol and re-suspended in 50 μl double-distilled water (ddW). The samples were solubilized in SDS-PAGE sample buffer without 2-mercaptoethanol. Equal amounts of samples (20 μl) were separated in 10 % SDS–PAGE containing gelatin (1 mg/ml) under non-reducing conditions. After electrophoresis, the gels were soaked in zymogram renaturing buffer (Invitrogen, Carlsbad, CA) for 60 min and incubated in zymogram developing buffer (Invitrogen) for 18 h at 37 °C. The gels were stained with 0.4 % Coomassie blue (Nacalai Tesque, Kyoto, Japan) for 15 min at room temperature and rapidly destained with destaining buffer (30 % methanol and 10 % acetic acid in ddW). Zones of proteolysis appeared as clear white bands against a blue background and were scanned using a Chemi DOC XRS system (Bio-Rad Laboratories). Band intensity was quantified using Quantity One software (Bio-Rad Laboratories).

### Statistical analysis

Data were expressed as means ± standard deviation (SD). A linear regression analysis was done using the method of least squares. Significance was analyzed by the Spearman’s rank test. Experiments with multiple comparisons were evaluated by one way analysis of variance (ANOVA) followed by Scheffé’s test to adjust for multiple comparisons. Graphpad Prism 5 (GraphPad Software, Inc., San Diego, CA) and StatView 4.54 (Abacus. Concepts, Inc., Berkeley, CA) were used for all analyses. Probability values of less than 0.05 were considered significant.

## Results

### Characteristics of the study subjects

The characteristics of the study subjects are listed in Table 1. Twenty-three control never-smokers, 17 control ex-smokers and 24 ex-smokers with COPD took part in the study. In the control never smoker group, more female subjects were included in this study compared to the other groups. There was no statistical difference in the parameters among the three groups except for lung function.

**Table 1:** Characteristics of the subjects in the lung tissue study

	Control never-smokers	Control ex-smokers	COPD ex-smokers
Subjects n	23	17	24
Male sex, %	30.4	82.4	87.5
Age, y	66.3 ± 9	65.3 ± 10	70.8 ± 7
Smoking status, pack-y	0	42.2 ± 20	49.0 ± 28
FEV_1,_ L	2.41 ± 0.60	2.55 ± 0.47	1.83 ± 0.53^**^
FEV_1_ %predicted, %	112 ± 16	92.5 ± 8.0^*^	72.7 ± 18^*, **^
FVC, L	3.05 ± 0.72	3.35 ± 0.66^*^	3.04 ± 0.74^**^
FVC %predicted, %	114 ± 15	98.8 ± 9.0	96.8 ± 17^*^
FEV_1_/FVC, %	79.2 ± 4.4	76.3 ± 4.1	59.6 ± 8.3^*, **^
D_LCO_/V_A_ %predicted, %^a^	96.1 ± 14	89.7 ± 15	79.9 ± 22^*^

Data are presented as mean ± SD. FEV_1_ = forced expiratory volume in 1 s; FVC = forced vital capacity; D_LCO_ = diffusing capacity of the lung for carbon monoxide; V_A_ = alveolar volume

^a^:D_LCO_/V_A_ are 23 healthy never-smokers, 16 healthy ex-smokers, and 22 COPD ex-smokers. ^*^: *p* < 0.05 compared to control never smokers. ^**^: *p* < 0.05 compared to control ex-smokers

### Analysis of KLF5 expression in the small airways and pulmonary vessels

**Fig. 1:**
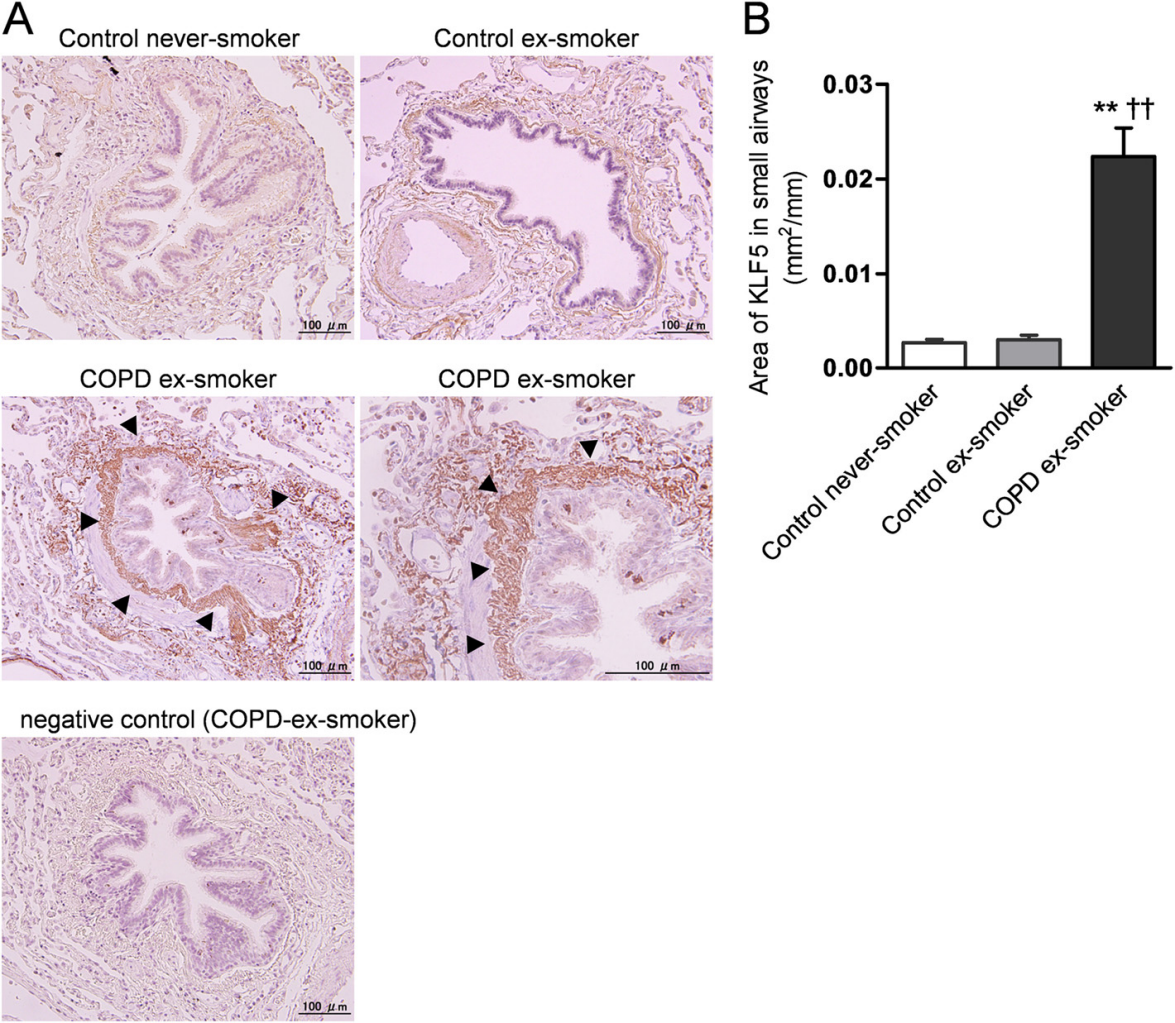
Semiquantitative measurement of Krüppel-like factor 5 (KLF5) production in the small airways by immunohistochemical staining. Lung tissues were obtained from control subjects that never or previously smoked and from former smokers with COPD. The localization of KLF5 in the lung tissues was investigated by immunostaining. Representative photographs of the immunoreactivity of KLF5 in the small airways are shown (original magnification × 200 for upper panels and left middle panel, × 400 for right middle panel; scale bars = 100 μm) (**a**). A negative control sample was provided to replace a non-specific polyclonal rabbit IgG antibody instead of the primary antibody on the lung tissue specimens of the patients with COPD. Arrow heads indicate KLF5-immunopositive cells. The immunopositive area of KLF5 in the small airways was quantified using Image J (**b**). **p < 0.01 *versus* control never smoker, ^††^ *p* < 0.01 *versus* control ex-smoker

**Fig. 2:**
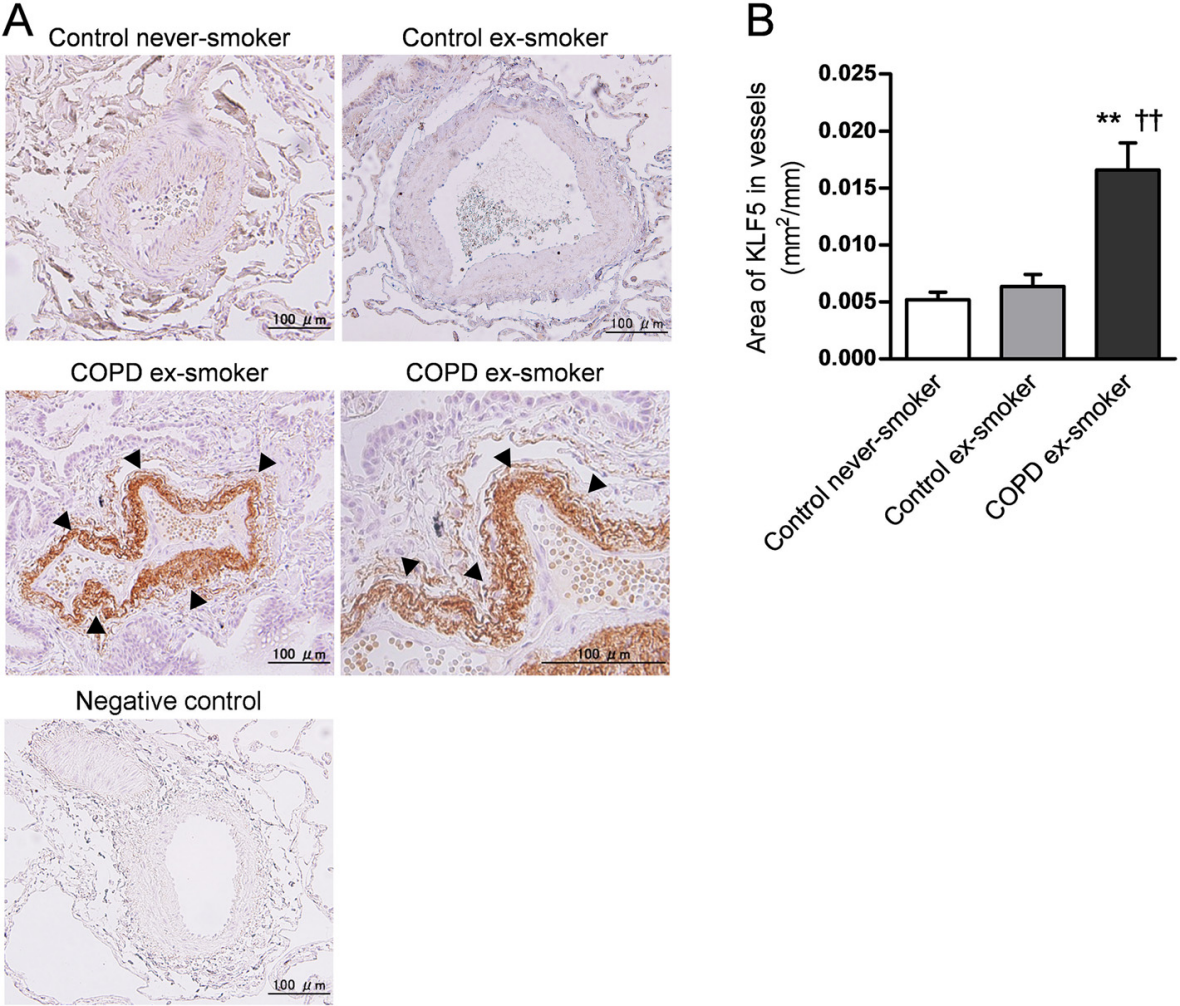
Semiquantitative measurement of KLF5 production in the pulmonary vessels by immunohistochemical staining. The localization of KLF5 in the lung tissues was investigated by immunostaining. Representative photographs of the immunoreactivity of KLF5 in the pulmonary vessels are shown (original magnification × 200 for upper panels and left lower panel, × 400 for right lower panel; scale bars = 100 μm) (**a**). A negative control sample was provided to replace the non-specific polyclonal rabbit IgG antibody instead of the primary antibody for the lung tissue specimens of the patients with COPD. Arrow heads indicate KLF5-immunopositive cells. The immunopositive area of KLF5 in the pulmonary vessels was quantified using Image J (**b**). **p < 0.01 *versus* control never smoker, ^††^ *p* < 0.01 *versus* control ex-smoker

**Fig. 3:**
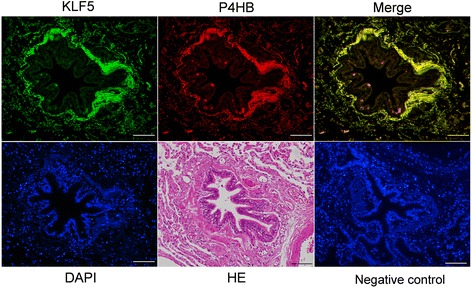
Immunoreactivity of KLF5 in the bronchial fibroblasts. To determine the cell types that expressed KLF5, double immunostaining for KLF5 and prolyl 4-hydroxylase β (P4HB) was carried out. P4HB is a marker of fibroblasts. Representative photographs of the immunoreactivity of KLF5 (green) and P4HB (red) are shown (original magnification × 200 for all panels; scale bars = 100 μm). A negative control sample was provided to replace the non-specific polyclonal rabbit IgG antibody instead of the primary antibody for the lung tissue specimens of the patients with COPD. DAPI: 4′,6-diamidino-2-phenylindole; HE: hematoxylin-eosin staining

**Fig. 4:**
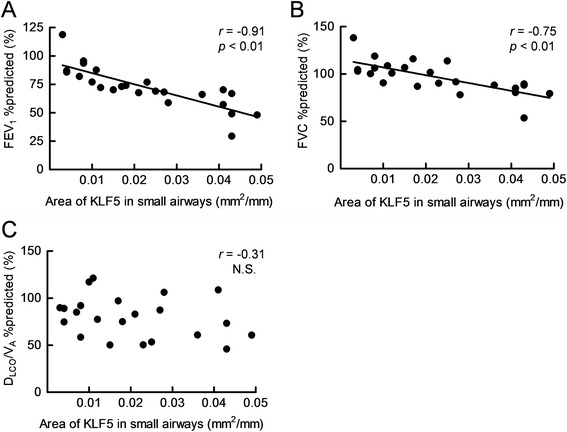
Relationship between the immunopositive area for KLF5 in the small airways and lung function. The relationship between the KLF5-positive area in the small airways and the values of FEV_1_ %predicted (**a**), FVC %predicted (**b**), and D_LCO_/V_A_ %predicted (**c**) was investigated in the COPD group. r is the correlation coefficient; the lines and p values correspond to the fitted regression equation. FEV_1_: forced expiratory volume in 1 s; FVC: forced vital capacity; D_LCO_: diffusing capacity of the lung for carbon monoxide; V_A_: alveolar volume; N.S.: not significant

We investigated KLF5 expression in the lungs by immunohistochemistry. KLF5 was mainly expressed in the submucosa of the small airways (Fig. 1a). The KLF5-immunopositive area in the COPD group was significantly increased compared to that in the two control groups (Fig. 1a and b). The cells of pulmonary vessels from the COPD patients also strongly expressed KLF5 compared to those from the control subjects (Fig. 2a and b). To identify the cell types that expressed KLF5, we conducted double immunofluorescent staining. KLF5-immunopositive cells in the small airways also expressed P4HB, which is known to be a marker of fibroblasts (Fig. 3). The values of the KLF5-immunopositive areas in the small airways of the COPD group were significantly correlated with those of forced expiratory volume in 1 s (FEV_1_) %predicted (*r* = −0.91, *p* < 0.01; Fig. 4a) and forced vital capacity (FVC) %predicted (*r* = −0.75, *p* < 0.01; Fig. 4b), but not with the diffusing capacity of the lung for carbon monoxide/alveolar volume (D_LCO_/V_A_) %predicted (Fig. 4c).

### H_2_O_2_ and ONOO^−^ augment production and activation of KLF5 in HFL-1 cells

**Fig. 5:**
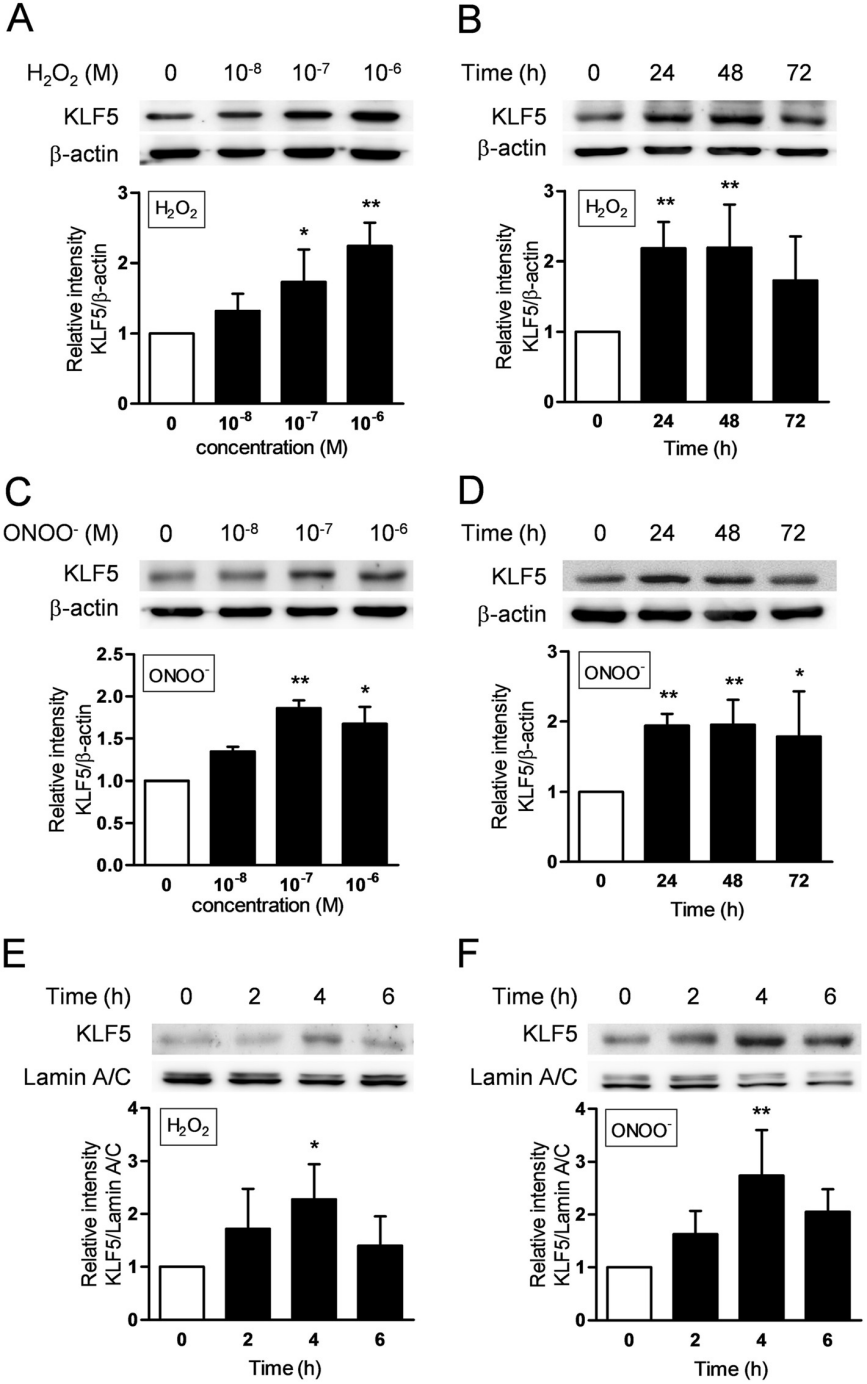
Effect of hydrogen peroxide (H_2_O_2_) or peroxynitrite (ONOO^−^) on KLF5 production and translocation into the nucleus in human fetal lung fibroblasts (HFL-1). Production of KLF5 was analyzed by immunoblotting. HFL-1 cells were treated with various concentrations of H_2_O_2_ for 24 h (**a**) and with 10^−6^ M H_2_O_2_ for various durations (**b**) as well as ONOO^−^ for 24 h (**c**) and 10^−7^ M ONOO^−^ for various durations (**d**). Each band intensity of KLF5 was standardized by that of β-actin. Translocation of KLF5 into the nucleus was also analyzed by immunoblotting. The cells were treated with 10^−6^ M H_2_O_2_ (**e**) or 10^−7^ M ONOO^−^ (**f**) and the nuclear fraction was harvested at various time points. Each band intensity of KLF5 was standardized by that of lamin A/C. All values are expressed as mean ± SD for 4–7 separate experiments. *p < 0.05, **p < 0.01 *versus* control group

To elucidate whether ROS and RNS augment the production of KLF5 in lung fibroblasts, HFL-1 were incubated with various concentrations of either H_2_O_2_ or ONOO^−^ and then the production of KLF5 was assessed by immunoblotting. H_2_O_2_ significantly augmented the production of KLF5 in a concentration-dependent manner compared to the control (at 10^−6^ M, 2.2-fold increase, *p* < 0.01, Fig. 5a). The production of KLF5 was significantly increased at 24 and 48 h after treatment with 10^−6^ M H_2_O_2_ (Fig. 5b). Similarly, ONOO^−^ significantly augmented the production of KLF5 compared to control (at 10^−7^ M, 1.9-fold increase, *p* < 0.01, Fig. 5c), and KLF5 was significantly increased at 24, 48 and 72 h after treatment with 10^−7^ M ONOO^−^ (Fig. 5d). Furthermore, we investigated the translocation of KLF5 into the nucleus to determine whether KLF5 was activated by ROS and RNS. After treatment with 10^−6^ M H_2_O_2_, the amount of KLF5 in the nuclear fraction was significantly increased at 4 h compared to control (at 4 h, 2.3-fold increase, *p* < 0.05, Fig. 5e) as well as ONOO^−^ (at 10^−7^ M, 4 h, 2.7-fold increase, *p* < 0.01, Fig. 5f).

### Effects of KLF5 silencing on the H_2_O_2_ or ONOO^−^-augmented production of α-SMA and release of collagens and MMPs

**Fig. 6:**
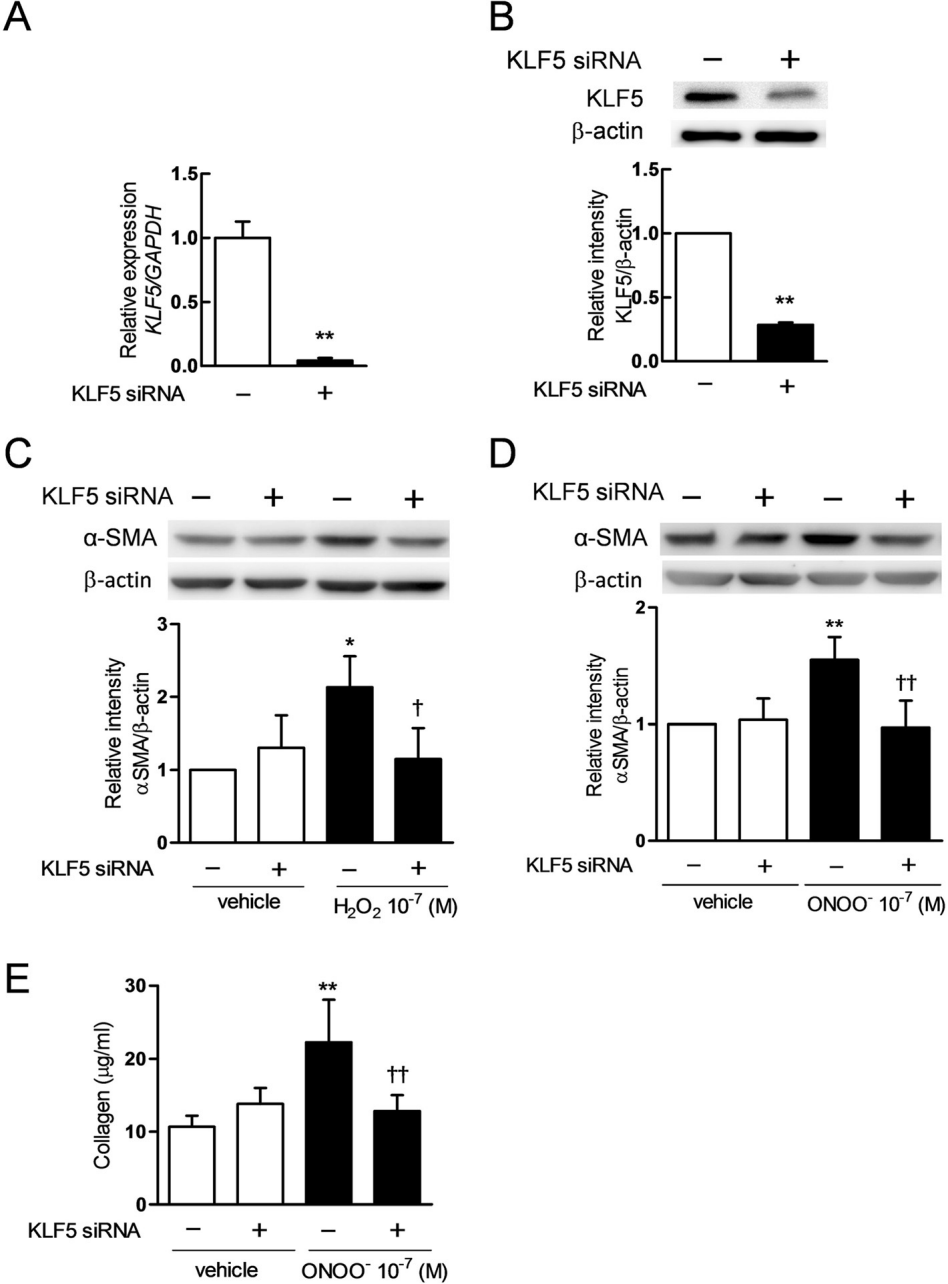
Effects of KLF5 silencing by siRNA on the H_2_O_2_ or ONOO^−^-augmented the production of α-smooth muscle actin (α-SMA) and release of collagens. Effects of siRNA for KLF5 on the gene expression and the protein production were confirmed by quantitative reverse transcription-polymerase chain reaction (qRT-PCR) for 24 h after transfection (**a**) and immunoblotting for 72 h (**b**). Effects of KLF5 silencing on the H_2_O_2_ (**c**) or the ONOO^−^ (**d**)-augmented production of α-SMA were analyzed by immunoblotting. Each band intensity was analyzed by Quantity One. Effect of KLF5 silencing on the ONOO^−^-augmented release of collagens was analyzed (**e**). All values are expressed as mean ± SD for 4 separate experiments. **p < 0.01 *versus* vehicle-pretreated vehicle-exposed group. ^†^ *p* < 0.05, ^††^ *p* < 0.01 *versus* vehicle-pretreated H_2_O_2_ or ONOO^−^-exposed group

**Fig. 7:**
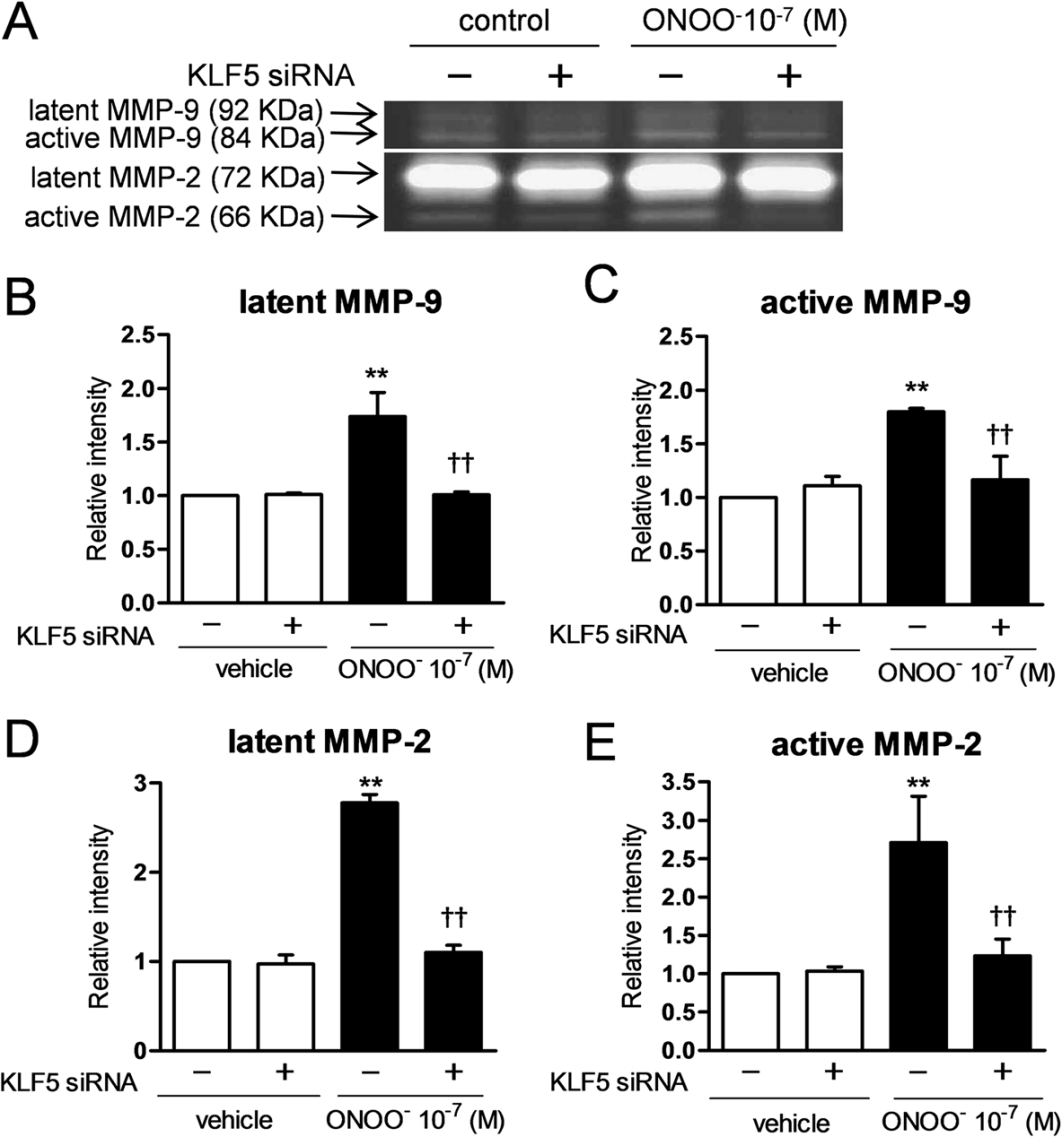
Effects of KLF5 silencing by siRNA on the ONOO^−^-augmented release of matrix metalloproteinases (MMPs). Effects of KLF5 silencing on the ONOO^−^-augmented release of MMP-9 and MMP-2 were investigated by gelatin zymography (**a**). After silencing, the infected cells were incubated with 10^−7^ M ONOO^−^ (filled bars) or vehicles (open bars) for 24 h. Latent form of MMP-9 (**a** and **b**), active form of MMP-9 (**a** and **c**), latent form of MMP-2 (**a** and **d**), and active form of MMP-2 were evaluated. Each band intensity was analyzed by Quantity One. All values are expressed as mean ± SD for 4 separate experiments. **p < 0.01 *versus* vehicle-pretreated vehicle-exposed group. ^†^ *p* < 0.05, ^††^ *p* < 0.01 *versus* vehicle-pretreated ONOO^−^-exposed group

To investigate whether KLF5 regulates the differentiation of fibroblasts into myofibroblast, the effect of KLF5 silencing on the H_2_O_2_ or ONOO^−^-augmented production of α-SMA was evaluated. The target siRNA diminished the mRNA expression and reduced the protein production of KLF5 (Fig. 6a and b). The siRNA significantly abrogated the H_2_O_2_ or ONOO^−^-augmented production of α-SMA (*p* < 0.01, Fig. 6c and d). The effect of KLF5 silencing on the release of collagen was also evaluated. The siRNA significantly abrogated the ONOO^−^-augmented release of collagen (*p* < 0.01, Fig. 6e). We investigated whether KLF5 regulated the fibroblast-mediated release of MMPs. The siRNA significantly abrogated the ONOO^−^ -augmented release of latent MMP-9 (*p* < 0.01, Fig. 7a and b), active MMP-9 (*p* < 0.01, Fig. 7a and c), latent MMP-2 (*p* < 0.01, Fig. 7a and d) and active MMP-2 (*p* < 0.01, Fig. 7a and e).

### Production of KLF5 in bronchial fibroblasts from the patients with COPD

**Fig. 8:**
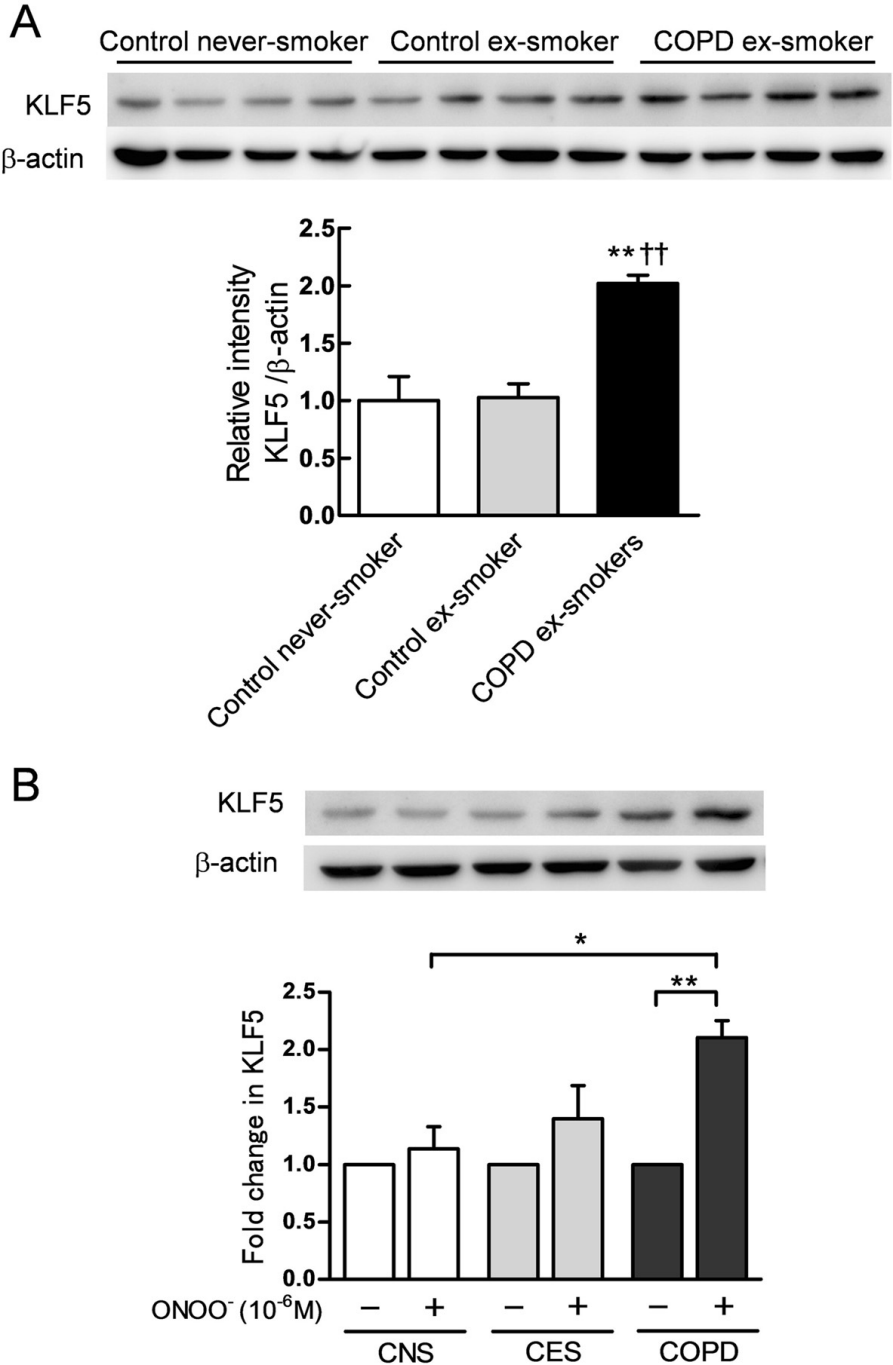
Production of KLF5 in adult human bronchial fibroblasts and effects of ONOO^−^ on production of KLF5 in adult cells. Bronchial fibroblasts were isolated from the lungs of the subjects in each study group. Four different strains of bronchial fibroblasts in each group were obtained. The amounts of KLF5 were analyzed by immunoblotting (**a**). **p < 0.01 *versus* control never-smoker, ^††^ *p* < 0.01 *versus* control ex-smoker. The fibroblasts were treated with 10^−6^ M ONOO^−^ for 24 h. The production of KLF5 was analyzed by immunoblotting (**b**). Each band intensity of KLF5 was standardized by that of β-actin. *p < 0.05, **p < 0.01. All values are expressed as mean ± SD. CNS = Control never-smoker; CES = Control ex-smoker, COPD = COPD ex-smoker

To confirm that bronchial fibroblasts from the COPD patients express more KLF5, we used cells from the control subjects and the age-matched patients with COPD (Table 2). Under the basal condition, the COPD cells showed significantly increased the production of KLF5 compared to those of the control subjects (*p* < 0.01, Fig. 8a). In the COPD cells, ONOO^−^ markedly stimulated production of KLF5 (*p* < 0.01, Fig 8b) and the fold changes of KLF5 in the COPD group significantly increased compared to those of the control never-smoker group (*p* < 0.05, Fig. 8b), suggesting that COPD bronchial fibroblasts were more susceptible to KLF5 expression induced by nitrosative stress compared to control cells.

**Table 2:** Characteristics of the subjects in the bronchial fibroblasts study

	Control never-smokers	Control ex-smokers	COPD ex-smokers
Subjects n	4	4	4
Age, y	61.5 ± 15	70.8 ± 3	67.0 ± 3
FEV_1,_ L	2.86 ± 0.93	2.65 ± 0.84	2.42 ± 0.51
FEV_1_ %predicted, %	110 ± 16	112 ± 18	83.6 ± 17 ^*,**^
FVC, L	3.46 ± 0.92	3.24 ± 1.2	3.72 ± 0.66
FEV_1_/FVC, %	81.8 ± 4.5	77.6 ± 7.9	63.8 ± 8.3 ^*,**^

Data are presented as mean ± SD. FEV_1_ = forced expiratory volume in 1 s; FVC = forced vital capacity

^*^: *p* < 0.05 compared to control never smokers. ^**^: *p* < 0.05 compared to control ex-smokers

## Discussion

In the present study, we demonstrated that the expression of KLF5 was up-regulated in the small airways and pulmonary small vessels of patients with COPD compared to those of control subjects, and that KLF5 was mainly expressed in the submucosal fibroblasts and cells of the pulmonary vessels in the patients with COPD. The expression levels in the small airways were significantly correlated with the severity of the airflow limitation. The *in vitro* study demonstrated that oxidative/nitrosative stress, which is a major cause of the pathogenesis in COPD, augmented the expression of KLF5 in the human lung fibroblasts as well as the translocation of KLF5 into the nuclei. Silencing of KLF5 significantly suppressed production of α-SMA, the release of collagen and MMPs from lung fibroblasts. The COPD bronchial fibroblasts expressed more KLF5 compared to those from the control subjects. In addition, the COPD fibroblasts had more susceptibility to KLF5 overexpression induced by nitrosative stress compare to that in control never-smokers. These results suggest that KLF5 may play a pivotal role in the tissue remodeling in patients with COPD.

Airway obstruction is commonly observed in the small airways of patients with COPD, and it is thought to be a major cause of the airflow limitation in patients with COPD as well as pulmonary emphysema [3]. Although some previous reports suggested that hyperproduction of TGF-β_1_ released from inflammatory cells could be involved in the airway remodeling [4–6], the possible mechanisms underlying small airway remodeling in COPD has remained poorly understood. KLF2, one of KLF family is considered to be involved in lung development. Among KLF families, KLF5 is reported to regulate embryonic development, cellular differentiation and proliferation [21–23]. Recently, it was reported that KLF5 is highly expressed in cardiac fibroblasts under pressure overload and contributes to the development of cardiac hypertrophy and fibrosis through the release of several growth factors including TGF-β, platelet-derived growth factor (PDGF), and insulin-like growth factors (IGF) [24, 25]. However, whether the expression of KLF5 occurs in the normal respiratory system as well as under pathological conditions is not known. In the current study, we first found increased expression of KLF5 in the fibroblasts of the small airways in the patients with COPD compared to the control subjects. Because KLF5 is thought to regulate tissue remodeling and fibrosis in other organs, KLF5 may be involved in the remodeling of small airways.

Interestingly, we found that the cells of the pulmonary arterial wall in the patients with COPD strongly expressed KLF5 compared to those of the control subjects, as shown in Fig. 2. Because the expression of KLF5 is reportedly up-regulated in the cells of arteries in the systemic circulation and regulates vascular remodeling [25], the strong expression of KLF5 in the pulmonary vessels of COPD patients may be involved in the remodeling of the pulmonary vessels. Recent findings have suggested that pulmonary hypertension (PH) plays a key role in the pathophysiology of COPD [27]. This hypothesis is supported by the observation that a large proportion of patients with COPD also have PH, with percentages ranging from 30 to 70 % [28], and that even smokers who do not suffer from COPD can develop pulmonary vascular remodeling [29]. A recent study also showed that the expression levels of KLF5 in pulmonary artery smooth muscle cells from patients with PH are higher than in those from healthy subjects [30]. These findings suggest that KLF5 could become a therapeutic target in COPD patients with PH.

We demonstrated in the present study that the degree of expression of KLF5 had a significantly positive correlation with the severity of the airflow limitation. Both obstruction of the small airways and easy collapsibility due to the loss of elastic recoil are believed to contribute to the airflow limitation in COPD. In this study, we could not evaluate the role of KLF5 in the loss of elastic recoil in the pulmonary parenchyma. However, Hogg JC et al*.* showed that the progression of COPD from mild to very severe was most strongly associated with thickening of the small airway walls and each of their compartments by a repair or remodeling process [3]. Furthermore, McDonough JE et al*.* demonstrated that the obstruction and disappearance of small conducting airways could initiate the onset of emphysematous destruction in COPD [31]. Because the COPD of the patients who enrolled in this study was relatively mild to moderate, the obstruction of the small airways might have had a stronger effect on the airflow limitation than the loss of elastic recoil.

We found that ROS and RNS are potent inducers and activators of KLF5 in human lung fibroblasts. KLF5 expression is induced by various stimuli in other cells. In vascular smooth muscle cells, KLF5 was reported to be up-regulated by angiotensin II *via* endogenous ROS [32, 33]. In addition, exogenous H_2_O_2_ augmented the mRNA expression of KLF5 in vascular smooth muscle cells [32]. These finding support our current data. The mechanism for the induction and activation of KLF5 by ROS and RNS should be clarified and the signal transduction for the regulation of KLF5 gene should be addressed in greater detail.

Oxidative stress is an important mechanism that accelerates the deterioration of lung function in COPD [16]. Many reports showed that oxidative stress activates inflammatory genes, inactivates antiproteases and stimulates mucus secretion [15, 16]. Nitrosative stress is induced by RNS such as ONOO^−^, and we first found that RNS production was enhanced in the airways of COPD patients [17]. According to the GOLD guidelines, RNS are considered to mediate many of the adverse effects of oxidative stress. Although endogenous or exogenously produced ROS and RNS have been clearly shown to possess many adverse effects involved in the pathogenesis of COPD, they might affect the development of airway remodeling *via* KLF5 pathway.

We clearly showed that KLF5 regulated the tissue remodeling responses including myofibroblast differentiation, and the release of collagen and MMPs from human lung fibroblasts, since the silencing of KLF5 gene abrogated the oxidative/nitrosative stress-augmented tissue remodeling responses. Previous reports showed that KLF5 regulated tissue remodeling through the release of several growth factors such as TGF, PDGF, and IGF [24, 25]. Furthermore, it has been reported that the overexpression of KLF5 caused gelatin degradation by stimulating the promoter activity of MMP-9 without affecting chondrocyte differentiation or vascular endothelial growth factor expression in cultured chondrogenic cells [34]. In addition, KLF5 dysfunction by genetic heterodeficiency or RNA interference was confirmed to cause a reduction of MMP-9 expression in cultured chondrogenic cells [34]. These findings support our current data. Our results suggest that KLF5 is a key regulator of tissue remodeling and closely involved in the pathogenesis of COPD.

In this study, we confirmed the overexpression of KLF5 in bronchial fibroblasts from the COPD patients compared to that from the control subjects under the basal condition. The cells from the COPD patients were more susceptible to KLF5 overexpression induced by nitrosative stress. In our previous reports, bronchial fibroblasts from asthmatic mice showed a profibrotic property compared to those from control mice [35], and the lung fibroblasts from severe COPD patients showed impaired tissue repair function [36]. Furthermore, we previously demonstrated that bronchial fibroblasts from asthmatic patients released more MMPs when the cells were stimulated by ligand for toll-like receptor 3 [37]. Little is known about whether there are differences in the susceptibility to stimuli which may possibly influence the development and progression of the pathological changes observed in the lungs of COPD paients.

There are several limitations in this study. First, both ROS and RNS are generally reported to stimulate tissue remodeling through NF-κB and/or AP-1. It is well known that, when these transcription factors are activated, tissue remodeling is accelerated *via* TGF-β and MMP production [18, 37]. We did not investigate the crosstalk between KLF5 and other transcription factors in the current study. Second, Takeda and coworkers demonstrated that cardiac pressure overload stimulated the expression of KLF5 in cardiac fibroblasts and that IGF-1 released from activated fibroblasts caused hypertrophy of the myocardium *in vivo* through crosstalk between myocardial fibroblasts and cardiac myocytes [24]. In the present study, we did not evaluate cell-cell interactions such as those between airway epithelial cells and fibroblasts. Further studies are needed to clarify whether KLF5 can interact with other transcription factors and other types of cells in the airways.

## Conclusions

The current study showed that the expression of KLF5 was enhanced in the peripheral airways and pulmonary vessels of patients with COPD compared to those of the control subjects. KLF5 was mainly expressed in the submucosal fibroblasts of small airways and the cells of pulmonary vessels. The expression levels were well correlated with the severity of the airflow limitation in COPD. Oxidative/nitrosative stress augmented the expression and activation of KLF5 in the human lung fibroblasts and KLF5 could regulate the fibroblast-mediated tissue remodeling. Bronchial fibroblasts from COPD patients were more susceptible to KLF5 overexpression against nitrosative stress. Our data suggest a possible mechanism in the KLF5-regulated tissue remodeling in the small airways and pulmonary vessels of COPD. Modulation of this pathway may have therapeutic potential for the tissue remodeling observed in patients with COPD.

## Abbreviations

AP-1: Activator protein-1
COPD: Chronic obstructive pulmonary disease
D_LCO_/V_A_: Diffusing capacity of the lung for carbon monoxide/alveolar volume
ECM: Extracellular matrix
FCS: Fetal calf serum
FEV_1_: Forced expiratory volume in 1 second
FVC: Forced vital capacity
GOLD: The Global Initiative for Chronic Obstructive Lung Disease
HFL-1: Human fetal lung fibroblasts
H_2_O_2_: Hydrogen peroxide
IGF: Insulin-like growth factors
KLF5: Krüppel-like factor 5
MMP: Matrix metalloproteinase
NF-κB: Nuclear factor-κB
PDGF: Platelet-derived growth factor
P4HB: Prolyl 4-hydroxylase
qRT-PCR: Quantitative reverse transcription-polymerase chain reaction
SD: Standard deviation
siRNA: Small interfering RNA
RNS: Reactive nitrogen species
ROS: Reactive oxygen species
TGF: Transforming growth factor
